# Structural analogs modulate olfactory and behavioral responses to a bile acid sex pheromone in sea lamprey (*Petromyzon marinus*)

**DOI:** 10.1007/s00359-025-01760-7

**Published:** 2025-09-27

**Authors:** Anne M. Scott, Ke Li, Joseph J. Riedy, Weiming Li

**Affiliations:** 1 Department of Fisheries and Wildlife, Michigan State University, 480 Wilson Rd, East Lansing, MI 48824 USA; 2 Present Address: Yantai Institute of Coastal Zone Research, Chinese Academy of Sciences, Shandong, 264003 China; 3 Present Address: Biological Sciences Program, Michigan State University, 642 Red Cedar Rd. #1110, East Lansing, MI 48824 USA

**Keywords:** Olfaction, Electrophysiology, Structure-activity relationships, Sensory ecology, Chemoreception, Behavioral antagonist

## Abstract

Male sea lamprey (*Petromyzon marinus*) release a sex pheromone featuring a potent bile acid 3-keto petromyzonol sulfate (3kPZS), which plays a critical role in attracting ovulated females to spawning sites during their terminal reproductive phase. In this study, we evaluated how systematic modifications of 3kPZS, referred to as the 11 analogs that retain the 3kPZS core structure with one or more functional group substitutions, affect female sea lamprey neurophysiological and behavioral responses to 3kPZS. Using electro-olfactogram recordings and two-choice flume behavioral assays, we characterized responses elicited by each analog and assessed whether the analogs interfered with 3kPZS-induced responses. Our results demonstrate that bile acid analogs with substitutions of hydroxyl or ketone groups with sulfate moieties at carbon positions 3, 7, 12, and 24 elicit distinct olfactory and behavioral responses in sea lamprey. Analogs with a sulfate group at the carbon-24 position tended to elicit potent olfactory responses of comparable magnitude to 3kPZS. Adding more sulfates at the carbon-3, 7, or 12 position altered behavioral valence and often neutralized or reversed female attraction to 3kPZS in a flume. These data elucidate structure-activity relationships and identify key structural determinants underlying sea lamprey olfactory detection and odorant-mediated behavioral responses. The findings may inform a potential approach for managing invasive sea lamprey populations in the Laurentian Great Lakes by disrupting bile acid mediated pheromone communication. Further research is needed to assess the utility of these compounds in natural stream environments and to refine the structural features of these pheromone antagonists to enhance their efficacy.

**Supplementary Information:**

The online version contains supplementary material available at 10.1007/s00359-025-01760-7.

## Introduction

**Fig. 1:**
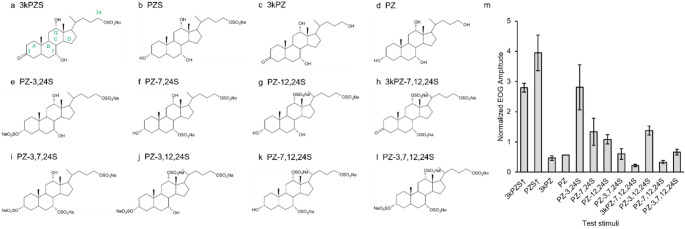
The chemical structures and electro-olfactogram (EOG) responses elicited by a main component of the male sea lamprey sex pheromone and 11 analogs. (A) 3-keto-petromyzonol-24-sulfate sodium salt (3kPZS), showing the four fused rings (designated A, B, C, and D) and canonical atom numbers for the steroid scaffold and tail in green; (B) Petromyzonol-24-sulfate sodium salt (PZS), (C) 3-keto-petromyzonol (3kPZ); (D) Petromyzonol (PZ); (E) Petromyzonol-3,24-disulfate disodium salt (PZ-3,24 S); (F) Petromyzonol-7,24-disulfate disodium salt (PZ-7,24 S); (G) Petromyzonol-12,24-disulfate disodium salt (PZ-12,24 S); (H) 3-keto-petromyzonol-7,12,24-trisulfate trisodium salt (3kPZ-7,12,24 S); (I) Petromyzonol-3,7,24-trisulfate trisodium salt (PZ-3,7,24 S); (J) Petromyzonol-3,12,24-trisulfate trisodium salt (PZ-3,12,24 S); (K) Petromyzonol-7,12,24-trisulfate trisodium salt (PZ-7,12,24 S); (L) Petromyzonol-3,7,12,24-tetrasulfate tetrasodium salt (PZ-3,7,12,24 S); S, sulfate. (M) The EOG response amplitude of each test stimuli at 10^− 6^ M was measured, blank-corrected, and normalized to the response amplitude of 10^− 5^ M L-arginine (standard). Reponses were measured in adult sea lamprey. Data are represented as mean ± SEM. *n* = 6 for all test stimuli, except *n* = 5 for 3kPZ and PZ, and *n* = 12 for PZ-3,7,12,24 S. † Data from Buchinger et al. 2020 used under CC BY-NC-ND 4.0 license

Bile acids and bile alcohols, collectively termed bile salts, are known to be potent odorants for numerous fish species (Hara 1994b; Døving et al. 1980). These steroids are synthesized in the liver from cholesterol and function primarily to emulsify dietary fats and aid in lipid absorption in the intestine of vertebrates (Hofmann et al. 2010). Bile salts have a core structure of seventeen carbons fused into a four-ring structure consisting of three cyclohexane rings (rings A-C) and one cylcopentane ring (ring D), together with either a five or eight carbon side chain that terminates in a carboxyl group (bile acids) or a primary alcoholic group (bile alcohols) and default hydroxylation at carbon-3 position (C-3) and C-7 (Hofmann et al. 2010; Fig. 1a). These combinations give rise to three classes of bile salts, including C_27_ bile alcohols, C_27_ bile acids, and C_24_ bile acids. Bile salts exhibit great structural diversity among taxa with variations based on the configuration of the A and B ring junction (5-α or 5-β), degree of saturation, presence of hydroxyl groups and their orientation, and addition of substituent groups either on a ring or side chain or both (Hofmann et al. 2010; Hagey et al. 2010). Secreted bile salts are reabsorbed from the distal small intestine and returned to the liver via enterohepatic circulation. However, bile salts are not completely reabsorbed by the intestine. In fish, the unabsorbed bile salts can be excreted in the urine, feces, or from the gills into the surrounding water (Huertas et al. 2010; Frade et al. 2002; Zhang et al. 2001; Li et al. 2002). Excreted bile salts are often conjugated with a sulfate group as bile alcohols or conjugated with a glycine, taurine, or taurine derivative as bile acids to increase solubility in aquatic environments. The functional groups present on bile salts also impact the olfactory sensitivity and specificity (Hara 1994a).

Like other odorants, bile salts are detected by olfactory receptors on olfactory sensory neurons in the olfactory epithelia. Fish olfactory systems are highly sensitive and specific to bile salts, capable of detecting and discriminating minor structural variations at sub-nanomolar concentrations (Zhang and Hara 2009; Li et al. 1995). The detection of olfactory cues allow fish to evaluate their environment and guides key behaviors including migration, foraging, assessing risk, and reproduction (Buchinger et al. 2014); however, the biological function underlying widespread fish olfactory sensitivity to bile acids remains poorly understood aside from the notable exception of sea lamprey (*Petromyzon marinus*) (Bjerselius et al. 2000; Li et al. 2002).

Sulfated bile acid derivatives have been identified as key components of the chemical communication system of sea lamprey, a basal vertebrate. Sea lamprey inhabit tributary streams as larvae, enter the Atlantic Ocean or large lakes to feed during a parasitic phase, and then return to streams as adults to spawn and die (Hardisty and Potter 1971). The composition and concentration of bile acids released by sea lamprey change at different life stages and induce characteristic behaviors in adults (Bjerselius et al. 2000; Li et al. 2002) that facilitate the identification of spawning streams and interaction with mates (Fissette et al. 2021). Stream-resident larval sea lamprey release bile acids that guide conspecific adults undertaking reproductive migration (Bjerselius et al. 2000). Anadromous adult sea lamprey do not home to their natal streams (Bergstedt and Seelye 1995), but instead recognize and select streams laden with the larval-released migratory cue (Bjerselius et al. 2000; Sorensen et al. 2003, 2005; Teeter 1980). The larval migratory cue consists of bile acid derivatives including petromyzonol sulfate (PZS), petromyzonamine disulfate (PADS), and petromyzosterol disulfate (PSDS) (Fine and Sorensen 2010) that induce electrophysiological responses in the olfactory epithelium (Li et al. 1995; Sorensen et al. 2005) and behavioral preference of migratory sea lamprey in a laboratory flume (Vrieze and Sorensen 2001; Sorensen et al. 2005; Bjerselius et al. 2000). Once in the river, adult sea lamprey sexually mature over several weeks into spermiated males and ovulated females. Sea lamprey form lek-like aggregations during spawning, where spermiated males construct nests in gravel substrate and release a multi-component sex pheromone consisting of bile acids that attracts ovulated females (Buchinger et al. 2015; Li et al. 2002). This pheromone-mediated mate attraction is critical in synchronizing spawning behaviors (Johnson et al. 2012). A main component of the male sex pheromone is 3-keto petromyzonol sulfate (3kPZS), which stimulates the olfactory epithelia of sea lamprey, increases ovulated female swimming activity, and induces mating related behaviors (Li et al. 2002; Siefkes et al. 2005; Johnson et al. 2009). In addition to 3kPZS, spermiated males release several other bile acid derivatives that may act as minor components of the sex pheromone (Li et al. 2013, 2017a, b, 2018b).

Studies of 3kPZS and PZS have shown that minor modifications of bile acids can drastically change their function as chemical cues. These two bile acids are detected by sea lamprey olfactory system with high sensitivity and activate at least two related odorant receptors (ORs), OR320a and OR320b (Zhang et al. 2020) but induce opposite behaviors. 3kPZS and PZS, which only differ in the hydrogenation of the oxygen at C-3, elicit similar olfactory potencies in the olfactory epithelium (Siefkes and Li 2004) and induce comparable activation of OR320a or OR320b receptors in vitro (Zhang et al. 2020). Additionally, some minor pheromone components that are also C_24_ 5α-bile acids activated OR320a and OR320b but with lower efficacy (Zhang et al. 2020). Despite similar chemical structures and olfactory potencies, 3kPZS and PZS induce different behavioral outcomes. PZS has been implicated in guiding migrating immature sea lamprey into rivers, whereas 3kPZS acts primarily as a male-released sex pheromone that attracts ovulated females. When tested in a two-choice flume, ovulated females preferred a channel activated with 3kPZS compared to vehicle but avoided PZS (Buchinger et al. 2020). Further, PZS antagonized the responses to 3kPZS in ovulated females. PZS reduced olfactory responses to 3kPZS and abated female preference for 3kPZS (Buchinger et al. 2020). Taken together, these findings show that two compounds that differ only by 2 hydrogens induce similar magnitude responses in cells expressing their cognate odorant receptors and in electrophysiology recordings of the olfactory epithelium, but induce opposite behaviors, highlighting the complexity of sea lamprey olfactory receptor-ligand interactions that ultimately dictate behavioral outcomes and potentially other downstream physiological processes.

The relationship between the chemical structure of a ligand and its biological activity is fundamental to chemical ecology. Structural modifications can drastically alter the way odorants interact with receptors, influencing olfactory responses and behavioral outcomes (Touhara and Vosshall 2009; Del Mármol et al. 2021). Structural modifications of bile acids such as substituting specific hydroxyls for sulfates appear to play an important role in modulating the activity of sea lamprey pheromones. Notably, replacing the hydroxyl on C-3, 7, and 12 of PZS with sulfate resulted in a derivate, petromyzonol-3,7,12,24-tetrasulfate tetrasodium salt (PZ-3,7,12,24 S), that synergized with PZS against 3kPZS-induced responses in ovulated female sea lamprey (Scott et al. 2023). This suggests that modifying bile acid sulfation patterns may alter known ligand interaction with olfactory receptors (PZS or 3kPZS with OR320a or OR320b), activate different olfactory receptors, or both, resulting in altered pheromone-induced olfactory and behavioral responses. Since sea lamprey are a destructive, invasive species in the Laurentian Great Lakes that contributed to the collapse of the Great Lakes fishery during the mid-20th century (Smith and Tibbles 1980; Coble et al. 1990) and necessitate ongoing control efforts, multiple studies have sought to identify compounds that disrupt their reliance on pheromone communication as potential supplemental control tactics (Scott et al. 2023; Buchinger et al. 2020; Raschka et al. 2018). A previous study screened approximately 300 commercially available analogs of PZS and 3kPZS and found the most active compounds that interfered with the olfactory detection of 3kPZS had a steroid scaffold and sulfate on the side chain (Raschka et al. 2018). However, because these studies were constrained to testing commercially available compounds, structural modifications were not systematically controlled, and multiple functional groups often varied simultaneously. As a result, it was difficult to isolate the specific role of individual chemical features, such as sulfate substitutions, in eliciting olfactory and behavioral responses in ovulated sea lamprey.

In this study, we systematically substitute the functional groups at C-3, 7, 12, and 24 on the bile acids 3kPZS and PZS with sulfate, allowing for a more precise comparative assessment of their influence on sea lamprey sensory and behavioral responses. Our results show that sulfate substitutions at specific positions within the bile acid scaffold alter olfactory responses and subsequent behavioral attraction to 3kPZS. Our findings contribute to a broader understanding of how structural analogs influence olfactory detection of 3kPZS that mediates chemosensory behaviors and offer insights into potential control strategies that manipulate olfactory cues to manage invasive sea lamprey in the Laurentian Great Lakes Basin.

## Methods

### Animals

Pre-spawning adult sea lamprey were captured by the U.S. Fish and Wildlife Service and Fisheries and Oceans Canada with scientific collection permits. Sea lamprey were transported to the U.S. Geological Survey, Hammond Bay Biological Station (HBBS), Millersburg, MI, USA and held in 200–1000 L flow-through tanks supplied with aerated, ambient Lake Huron water. Pre-spawning adult sea lamprey used for EOG recordings were transported to Michigan State University, East Lansing, MI, USA and held in 250 L flow-through tanks supplied with aerated water maintained at 7 to 9 °C. All behavioral experiments used sexually mature female (ovulated) sea lamprey that were produced by holding pre-spawning female sea lamprey in the lower Ocqueoc River, Presque Isle County, MI, USA. Fish were assessed daily for expression of gametes (Siefkes et al. 2003; Johnson et al. 2015). Once sexually mature, ovulated female sea lamprey were returned to HBBS and held in 200 L flow-through, aerated tanks until tested in behavioral assays.

### Stimuli preparation

The odorant stimuli include 3-keto-petromyzonol-24-sulfate sodium salt (3kPZS, Bridge Organics Co., Vicksburg, MI, USA, Lot # 180-EJH-290-3), petromyzonol-24-sulfate sodium salt (PZS, Bridge Organics Co., Lot # 248-EJH-60-2), 3-keto-petromyzonol (3kPZ, Cayman Chemical Co., Ann Arbor, MI, USA, Catalog # 10007055, Lot # 0417187-13), petromyzonol (PZ, Cayman Chemical Co., Catalog # 98250, Lot # 0550235-5). The following compounds were custom synthesized (Bridge Organics Co.) and not listed as commercially available in chemical databases (e.g., SciFinder): petromyzonol-3,24-disulfate disodium salt (PZ-3,24 S, Lot # 230-EJH-145-1), petromyzonol-7,24-disulfate disodium salt (PZ-7,24 S, Lot # 229-NAW-281), petromyzonol-12,24-disulfate disodium salt (PZ-12,24 S, Lot # 230-EJH-151-2), 3-keto-petromyzonol-7,12,24-trisulfate trisodium salt (3kPZ-7,12,24 S, Lot # 230-EJH-150-5), petromyzonol-3,7,24-trisulfate trisodium salt (PZ-3,7,24 S, Lot # 229-NAW-291), petromyzonol-3,12,24-trisulfate trisodium salt (PZ-3,12,24 S, Lot # 230-EJH-151-1), petromyzonol-7,12,24-trisulfate trisodium salt (PZ-7,12,24 S, Lot # 230-EJH-145-2), and petromyzonol-3,7,12,24-tetrasulfate tetrasodium salt (PZ-3,7,12,24 S, Lot # 229-NAW-290). 3kPZS, PZ-3,24 S, PZ-7,24 S, PZ-12,24 S, 3kPZ-7,12,24 S, PZ-3,7,24 S, PZ-3,12,24 S, PZ-7,12,24 S, and PZ-3,7,12,24 S (~ 10 mg each) was dissolved in the initial mobile phase of liquid chromatography and subjected to semi-preparative high performance liquid chromatography (HPLC) following established methods (Li et al. 2018b). Semi-preparative HPLC (Waters 1525 binary HPLC pump, 2996 photodiode array detector, and fraction collector III) was performed with a Luna RP-18 column (250 × 10 mm i.d.; 5 μm; Phenomenex), eluted with methanol and deionized water with a flow rate of 3.0 mL/min at room temperature. A gradient of methanol in deionized water from 30 to 100% over 30 min was used. Each compound was further purified with successive size exclusion chromatography via gel filtration on Sephadex LH-20 column (GE Healthcare, column size adjusted based on sample amount) described in Ó’Fágáin (2016) following procedures used by Li et al. (2018a) with a mobile phase of 100% methanol and methanol: dichloromethane mixture (1:1, v: v). The purity of all compounds was confirmed to be ≥ 95%. The purity of 3kPZS was determined by comparison of HPLC results with that of a 3kPZS standard. Since standards were not commercially available for the sulfated petromyzonol compounds, the purity of these compounds was evaluated by HPLC area normalization procedures as described in Épshtein (2004). Then, 10^− 3^ M stock solutions of each purified stimulus in water: methanol (1:1, v: v) were prepared and stored at -20 °C until experimentation. A 10^− 2^ M stock solution of L-arginine (Sigma-Aldrich, St. Louis, MO, USA, Catalog # A5006) in deionized water was prepared and stored at 4 °C until experimentation.

### Electro-olfactogram (EOG) setup and recordings

EOG recordings followed previously established procedures (Li et al. 2018a; Scott et al. 2019) to measure olfactory responses after exposure to odorants in May – July 2013, 2016, 2017, and 2021. Sea lamprey were anesthetized with ethyl 3-aminobenzoate (100 mg L^− 1^, MS222, Sigma-Aldrich) and immobilized with an injection of gallamine triethiodide (30 mg kg^− 1^ of body weight). The anesthetized fish was oriented on a plastic stand and wrapped with an absorbent towel to prevent desiccation in a water filled trough with the head of the lamprey remaining above the water. Gills were continuously irrigated with aerated water containing 50 mg L^− 1^ MS222 throughout the duration of the experiment. The olfactory lamellae were exposed, perfused with water, and the differential EOG response was recorded using glass capillaries filled with 0.4% agar in 0.9% saline that were connected to solid state electrodes with Ag/AgCl pellets model (ESP-M15N; Warner Instruments, Hamden, CT, USA) in 3 M KCl. The recording electrode was positioned near the ventral ridge between two olfactory lamellae and adjusted to maximize the response to L-arginine standard while minimizing the response to the blank control (charcoal filtered water). The reference electrode was positioned on the surface of the skin near the naris. EOG signals were amplified by a NeuroLog system (model NL102; Digitimer, Welwyn Garden City, England, UK), filtered with a lowpass 60 Hz filter (model NL125; Digitimer), digitized by Digidata 1440 A (Molecular Devices, San Jose, CA, USA), and recorded on a PC running AxoScope 10.4 software (Molecular Devices).

To record responses to each analog, 10^− 3^ M stock solutions of each analog were diluted with filtered water resulting in 10^− 6^ M test solutions. A 10^− 2^ M stock solution of L-arginine in deionized water was diluted to 10^− 5^ M test solution on the day of the experiment and applied to the olfactory epithelium for 4 s. The response to L-arginine 10^− 5^ M was recorded to normalize for variations in olfactory sensitivity among individual sea lamprey. The olfactory epithelium was flushed with filtered water for 2 min, the blank control (background water) introduced, and the response recorded. Then, 10^− 6^ M analog was applied, recorded, and flushed. A response to the blank control and 10^− 5^ M L-arginine standard were measured repeatedly after approximately every three stimuli throughout each recording session. The EOG response magnitudes were measured in millivolts (mV). The normalized EOG response was calculated as:

$$\:\text{N}\text{o}\text{r}\text{m}\text{a}\text{l}\text{i}\text{z}\text{e}\text{d}\:\text{E}\text{O}\text{G}\:\text{A}\text{m}\text{p}\text{l}\text{i}\text{t}\text{u}\text{d}\text{e}\:=\:\frac{\text{R}\text{t}-\text{R}\text{b}}{\text{R}\text{a}-\text{R}\text{b}}$$

where *Rt* is the response magnitude to the test stimulus, *Rb* is the response magnitude to the blank, and *Ra* is the response magnitude to L-arginine.

**Fig. 2:**
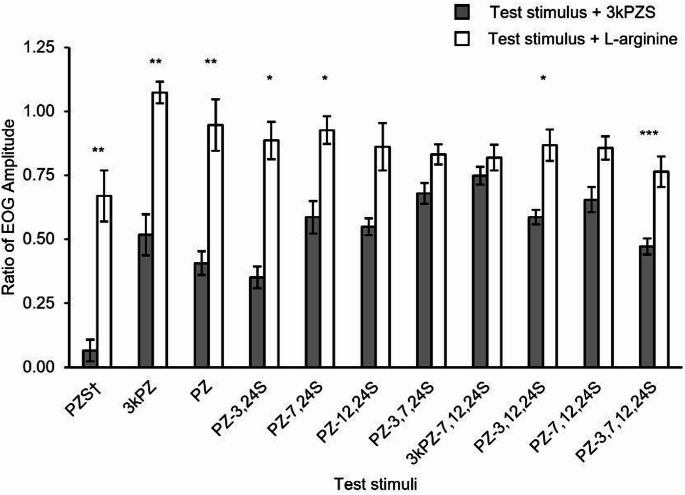
Analogs suppress electro-olfactogram (EOG) responses to stimuli measured in adult sea lamprey. Exposure to some analogs at 10^− 6^ M reduced EOG responses to 10^− 6^ M 3kPZS, but not to a non-pheromone control stimulus that elicits olfactory responses, 10^− 5^ M L-arginine. The response to 3kPZS or L-arginine during exposure to each analog was expressed as a ratio of EOG amplitude to 10^− 6^ M 3kPZS or 10^− 5^ M L-arginine during analog exposure versus before. A ratio of EOG amplitude value of 1.0 indicates the analog did not reduce the olfactory response to the test stimuli. Data are represented as mean ± SEM. The significance between mixtures within each analog was evaluated with paired two-tailed t test and Benjamini-Hochberg p value adjustment. * *P* < 0.05, ** *P* < 0.01, *** *P* < 0.001. *n* = 6 for all test stimuli, except *n* = 5 for 3kPZ and PZ, and *n* = 12 for PZ-3,7,12,24 S. † Data from Buchinger et al. 2020 used under CC BY-NC-ND 4.0 license

To evaluate whether analogs are likely to share olfactory detection mechanisms with 3kPZS and therefore reduce the 3kPZS olfactory response, we measured the 3kPZS EOG responses before and during exposure of the olfactory epithelium to each analog following established modified cross-adaptation approaches (Scott et al. 2019, 2023). Using this approach, if the response to 3kPZS is significantly reduced under the background of an analog, this indicates that 3kPZS and the analog likely interact with a common receptor/receptors and/or transduction mechanism. The EOG responses to 10^− 6^ M 3kPZS, 10^− 5^ M L-arginine, and blank were recorded. The naris was continuously exposed to 10^− 6^ M analog for 2 min. Next, the EOG responses to a mixture of 10^− 6^ M analog and 10^− 6^ M 3kPZS was recorded. The naris was rinsed for 2 min, and the responses to 3kPZS, L-arginine, and blank were measured. The change in 3kPZS olfactory response during exposure to analog was calculated as the ratio of the 3kPZS response during versus before analog exposure, defined as:

$$\:\text{R}\text{a}\text{t}\text{i}\text{o}\:\text{o}\text{f}\:\text{E}\text{O}\text{G}\:\text{A}\text{m}\text{p}\text{l}\text{i}\text{t}\text{u}\text{d}\text{e}=\:\left(\frac{\text{R}\text{d}\text{u}\text{r}\text{i}\text{n}\text{g}-\text{R}\text{b}}{\text{R}\text{a}-\text{R}\text{b}}\right)/\left(\frac{\text{R}\text{b}\text{e}\text{f}\text{o}\text{r}\text{e}-\text{R}\text{b}}{\text{R}\text{a}-\text{R}\text{b}}\right)$$

where *R*_*during*_ is the response to the mixture of 10^− 6^ M analog and 10^− 6^ M 3kPZS, *R*_*before*_ is the initial response to 10^− 6^ M 3kPZS, *Rb* is the response magnitude to the blank, and *Ra* is the response magnitude to 10^− 5^ M L-arginine. A ratio of EOG amplitude value of 1.0 indicates the analog did not reduce the olfactory response of 3kPZS, whereas a value of 0 indicates the analog reduced 100% of the olfactory response of 3kPZS. The experiment and analysis were repeated to determine if exposure to 10^− 6^ M analog reduced olfactory responses to 10^− 5^ M L-arginine, a non-pheromone control stimulus that elicits olfactory responses. Our previously published electro-olfactogram data for 3kPZS and PZS from Buchinger and colleagues (2020) were included under CC BY-NC-ND 4.0 license (https://creativecommons.org/licenses/by-nc-nd/4.0/) for comparison in Figs. 1m and 2, and Table 1.

### Two-choice flume assay

**Fig. 3:**
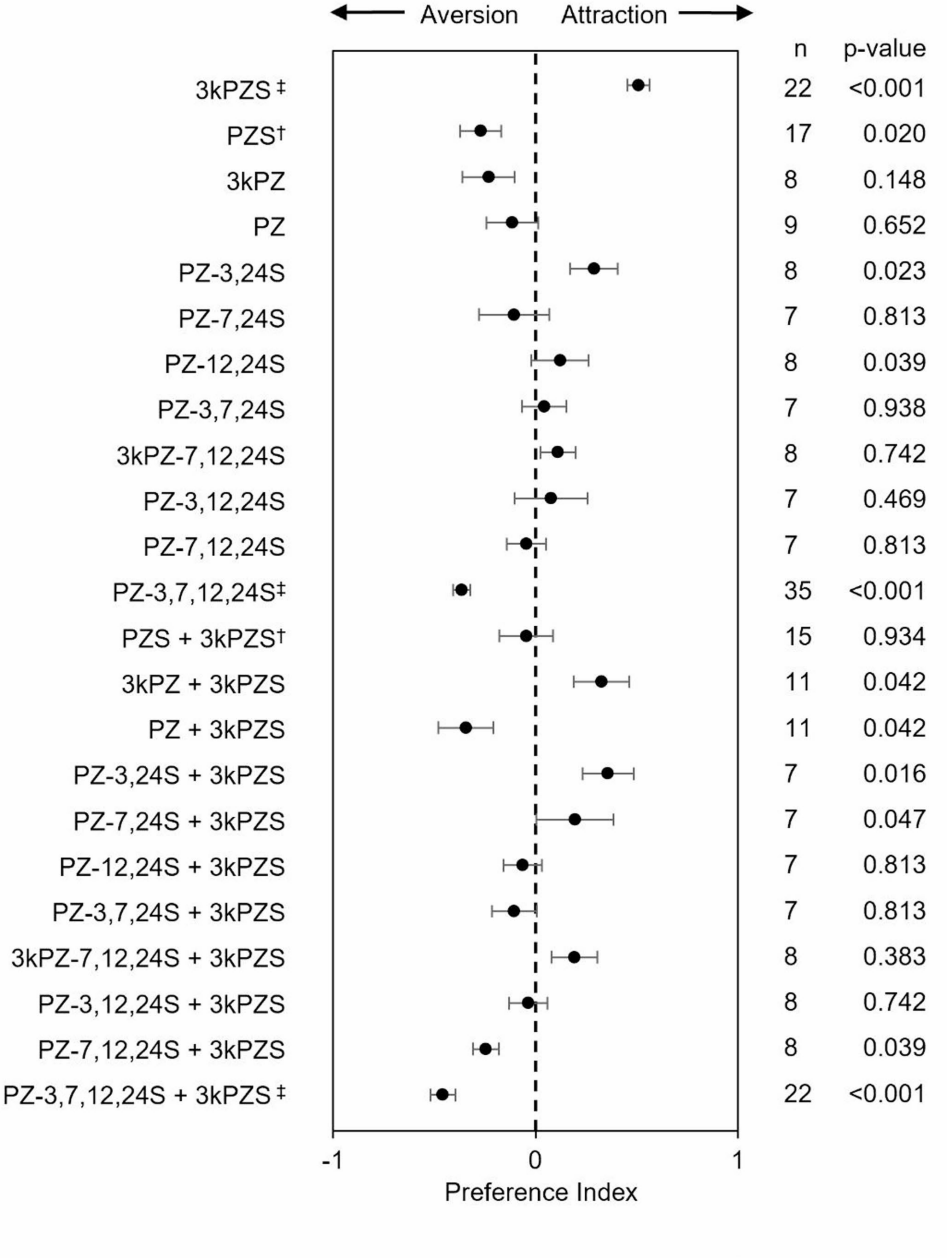
Behavioral responses of ovulated female sea lamprey in a two-choice flume. The preference index was calculated for each stimulant as the value of [Ae/(Ae + Be) − Ac/(Ac + Bc)], where Bc is the cumulative amount of time spent in the control channel before odorant application, Be is the cumulative amount of time spent in the experimental channel before odorant application, Ac is the cumulative amount of time spent in the control channel after odorant application, and Ae is the cumulative amount of time spent in the experimental channel after odorant application in a flume. Data are represented as mean ± SEM. Stimulants were applied to reach a concentration in the flume of 10^− 12^ M. A Wilcoxon signed-rank test was used to determine whether the mean index was different from zero. A positive index value indicates attraction, and a negative index value indicates aversion. 3kPZS attracted ovulated females and some of the analogs antagonized female attraction to 3kPZS by either neutralizing or repulsing them from experimental channel activated with 3kPZS in a two-choice flume. n, sample size, followed by the *P* value, is displayed (*Right*). † Data from Buchinger et al. 2020. ‡ Data from Scott et al. 2023

We used two-choice flumes to assay the behavioral responses of ovulated female sea lamprey to test stimuli in June – August 2013–2017 with water supplied from the Little Ocqueoc River (Presque Isle County, MI, USA) and in July – August 2021 with water supplied from Silver Creek (Presque Isle County, MI, USA) following established approaches (Buchinger et al. 2020; Scott et al. 2023). Both rivers are in the upper Ocqueoc River watershed, a historical sea lamprey spawning ground. However, a migration barrier now restricts sea lamprey from accessing the upper reaches. Therefore, no sea lamprey pheromone was present in the background water used in our behavioral assay. A sea lamprey was placed in an acclimation cage at the downstream end of the flume for 5 min, released, and the cumulative time the sea lamprey spent in each channel was recorded for 10 min (pre-treatment period before odorant application). Then, the test stimulus (3kPZS, analog, or mixture thereof) was introduced to a randomly chosen channel and vehicle (methanol: water, 1:1) to the other channel using a peristaltic pump at a rate of 200 ± 5 mL min^− 1^ for 5 min to reach a concentration in the flume of 10^− 12^ M. Then, the cumulative time the sea lamprey spent in each channel was recorded for 10 min while continuing to apply the treatment (odorant application period). The time spent in the control (*Bc*) and experimental (*Be*) channel before odorant application and in the control (*Ac*) and experimental (*Ae*) channel after odorant application were used to calculate a preference index (Li et al. 2002) for each trial as defined by:

$$\:\text{P}\text{r}\text{e}\text{f}\text{e}\text{r}\text{e}\text{n}\text{c}\text{e}\:\text{I}\text{n}\text{d}\text{e}\text{x}=\:\frac{\text{A}\text{e}}{(\text{A}\text{e}+\text{B}\text{e})}-\:\frac{\text{A}\text{c}}{(\text{A}\text{c}+\text{B}\text{c})}$$

which assessed the change in behavior following odorant application. Our previously published preference index data for 3kPZS, PZ-3,7,12,24 S, and a mixture of PZ-3,7,12,24 S and 3kPZS from Scott and colleagues (2023) under CC BY-NC-ND 4.0 license (https://creativecommons.org/licenses/by-nc-nd/4.0/) and PZS and mixture of PZS and 3kPZS from Buchinger and colleagues (2020) under CC BY-NC-ND 4.0 license (https://creativecommons.org/licenses/by-nc-nd/4.0/) were included for comparison in Fig. 3; Table 1.

### Quantification and statistical analysis

Data in plots represent the mean and standard error of the mean (SEM) denoted by the error bars. Statistical analyses were performed in R version 4.3.1. The response to 3kPZS or L-arginine during exposure to each analog was expressed as a ratio of EOG amplitude to 10^− 6^ M 3kPZS or 10^− 5^ M L-arginine during analog exposure versus before. A ratio of EOG amplitude value of 1.0 indicates that the analog did not reduce the olfactory response to the test stimuli. The difference in ratio of EOG amplitude values to mixtures within each analog was evaluated with paired two-tailed t test with a Benjamini-Hochberg p value adjustment to determine if the analog reduced response to 3kPZS more than those to L-arginine.

For two-choice flume assays, a trial was discarded if the sea lamprey failed to enter the control and experimental channel for at least 10 s during the 10-minute period before the odorant was applied as this was an indication of strong side bias or inactivity. A preference index for each test stimulus was evaluated using a Wilcoxon signed-rank test to determine if the index was significantly different from zero. A significant positive value of the preference index indicated attraction, and a significant negative preference index indicated aversion.

## Results

### EOG responses

Electro-olfactogram responses elicited by 10^− 6^ M 3kPZS and 11 analogs are shown in Fig. 1. All stimuli induced olfactory responses but to varying degrees. We discerned the structural features of bile acids that influence the magnitude of responses elicited at the olfactory epithelium via a comparative structure-activity analysis (Table 1). 3kPZS, PZS, and PZ-3,24-S were the most stimulatory test stimuli and induced response magnitudes approximately two-fold larger than all other analogs. These bile acids share three common features, namely a hydroxyl at C-7, hydroxyl at C-12, and sulfate at C-24. For analogs sharing identical functional groups at C-7, C-12, and C-24, replacing the C-3 hydroxyl with a ketone or sulfate group reduced the stimulatory effectiveness (PZS vs. 3kPZS; PZ vs. 3kPZ; PZ-7,12,24 S vs. 3kPZ-7,12,24 S). For analogs sharing identical functional groups at C-3, C-7, and C-12, removing the terminal sulfate group at C-24 and replacing it with a hydroxyl diminished the olfactory response (3kPZS vs. 3kPZ; PZS vs. PZ). For the analogs sharing identical functional groups at C-3, C-7, and C-24, those with a C-12 hydroxyl group often induced larger magnitude EOG responses than the analogs with a C-12 sulfate (PZS vs. PZ-12,24 S; PZ-3,24-S vs. PZ-3,12,24 S; PZ-7,24 S vs. PZ-7,12,24 S; except PZ-3,7,12,24 S vs. PZ-3,7,24 S). Broadly, bile acids bearing sulfate at C-7 and either C-3 or C-12 tended to elicit lower magnitude olfactory responses compared to other analogs (PZ-3,7,12,24 S, PZ-3,7,24 S, PZ-7,12,24 S, and 3kPZ-7,12,24 S).

**Table 1:** Summary of structure-activity relationship at carbon positions 3, 7, 12, and 24 of 3kPZS analogs

							Ratio of EOG amplitude		Behavioral preference index	
Name	Abbreviation	C-3	C-7	C-12	C-24	Normalized EOG amplitude at 10^− 6^ M	10^− 6^ M Stimulus + 10^− 6^ M 3kPZS	10^− 6^ M Stimulus + 10^− 5^ M L-arginine	10^− 12^ M Stimulus	10^− 12^ M Stimulus + 10^− 12^ M 3kPZS
3-keto-petromyzonol-24-sulfate sodium salt	3kPZS	=O	OH	OH	OSO_3_	2.80^†^	-	-	0.444^‡^	-
Petromyzonol-24-sulfate sodium salt	PZS	OH	OH	OH	OSO_3_	3.95^†^	0.07^†^	0.67^†^	-0.271^†^	-0.046^†^
3-keto-petromyzonol	3kPZ	=O	OH	OH	OH	0.46	0.52	1.07	-0.231	0.328
Petromyzonol	PZ	OH	OH	OH	OH	0.57	0.41	0.95	-0.113	-0.343
Petromyzonol-3,24-disulfate disodium salt	PZ-3,24 S	OSO_3_	OH	OH	OSO_3_	2.81	0.35	0.89	0.289	0.358
Petromyzonol-7,24-disulfate disodium salt	PZ-7,24 S	OH	OSO_3_	OH	OSO_3_	1.34	0.59	0.93	-0.105	0.195
Petromyzonol-12,24-disulfate disodium salt	PZ-12,24 S	OH	OH	OSO_3_	OSO_3_	1.08	0.55	0.86	0.122	-0.062
3-keto-petromyzonol-7,12,24-trisulfate trisodium salt	3kPZ-7,12,24 S	=O	OSO_3_	OSO_3_	OSO_3_	0.22	0.75	0.82	0.112	0.193
Petromyzonol-3,7,24-trisulfate trisodium salt	PZ-3,7,24 S	OSO_3_	OSO_3_	OH	OSO_3_	0.60	0.68	0.83	0.044	-0.104
Petromyzonol-3,12,24-trisulfate trisodium salt	PZ-3,12,24 S	OSO_3_	OH	OSO_3_	OSO_3_	1.38	0.59	0.87	0.078	-0.036
Petromyzonol-7,12,24-trisulfate trisodium salt	PZ-7,12,24 S	OH	OSO_3_	OSO_3_	OSO_3_	0.33	0.65	0.86	-0.044	-0.246
Petromyzonol-3,7,12,24-tetrasulfate tetrasodium salt	PZ-3,7,12,24 S	OSO_3_	OSO_3_	OSO_3_	OSO_3_	0.67	0.47	0.76	-0.349^‡^	-0.457^‡^

Electro-olfactogram recordings were used to measure olfactory response of each analog (Fig. 1) and the ratio of the 3kPZS response amplitude during analog exposure (Fig. 2). Behavioral assays were used to measure ovulated female preference for each analog and a mixture of each analog with 3kPZS (Fig. 3). † Data from Buchinger et al. 2020 used under CC BY-NC-ND 4.0 license. ‡ Data from Scott et al. 2023 used under CC BY-NC-ND 4.0 license

### Analog suppression of EOG response to 3kPZS

To investigate whether different analogs are likely to share olfactory detection mechanisms with 3kPZS, we used a modified cross-adaptation approach to assess how continuous exposure to each analog influenced the EOG response to 3kPZS. Compounds with hydroxyl at C-7, hydroxyl at C-12, and sulfate at C-24 (PZS and PZ-3,24-S) reduced the 3kPZS EOG response more than the other analogs tested (93 and 65% reduction, respectively; Fig. 2), suggesting these are three critical moieties for interacting with 3kPZS olfactory receptors. Analogs such as PZ and 3kPZ with a hydroxyl at C-7, hydroxyl at C-12, and non-sulfate functional group at C-24 were still quite effective at reducing the 3kPZS olfactory response (58 and 49% reduction, respectively; Fig. 2). This suggests that hydroxyl groups at C-7 and C-12 are important structural features for interacting with 3kPZS olfactory receptors, and the presence of a sulfate at C-24 further enhances this interaction. For pairs of analogs sharing identical functional groups at C-7, C-12, C-24, those that have a hydroxyl at C-3 reduced the 3kPZS olfactory response more than a sulfate (PZS vs. PZ-3,24 S; PZ-12,24 S vs. PZ-3,12,24 S; PZ-7,24 S vs. PZ-3,7,24 S) or ketone (PZ vs. 3kPZ; PZ-7,12,24 S vs. 3kPZ-7,12,24 S) (Table 1). PZS, 3kPZ, PZ, PZ-3,24 S, PZ-7,24 S, and PZ-3,12,24 S reduced the olfactory response to 3kPZS more than the olfactory response to L-arginine, suggesting that these analogs did not broadly suppress all olfactory responses to both bile acids (e.g., 3kPZS) and amino acids (e.g., L-arginine) but instead interact with a 3kPZS receptor and/or transduction mechanism (paired two-tailed t test, Benjamini-Hochberg adjusted *P* < 0.05; Fig. 2). In general, analogs that were effective in disrupting 3kPZS olfactory responses tended to have a sulfate at C-24, hydroxyl at C-12, hydroxyl at C-7, but the functional group at C-3 (hydroxyl, ketone, or sulfate) does not appear to influence efficacy of the analog as much.

### Behavioral responses to pheromone analogs

EOG recordings indicate whether analogs are odorants detected by the olfactory system and if the detection mechanism likely overlaps with that of 3kPZS but does not indicate the behavioral valence (i.e., analog induces attraction or aversion alone or when mixed with 3kPZS). Therefore, we characterized ovulated female preference for each analog alone (10^− 12^ M) and mixed with 3kPZS (1:1, each at 10^− 12^ M) in a flume (Fig. 3; Table 1). Ovulated females spent proportionally more time in the channel activated with 3kPZS when applied to produce a final concentration in the flume of 10^− 12^ M, compared to a channel treated with the vehicle (50% methanol) in a two-choice flume assay supplied with natural stream water (*P* < 0.001). PZ-3,24 S and PZ-12,24 S also attracted females. 3kPZS and PZ-3,24 S both share hydroxyls at C-7 and C-12 and sulfate at C-24. 3kPZ, PZ, PZ-7,24 S, PZ-3,7,24 S, PZ-3,12,24 S, PZ-7,12,24 S, 3kPZ-7,12,24 S did not induce a significant preference. Previous work by Buchinger et al. (2020) and Scott et al. (2023) showed ovulated females avoided PZS and PZ-3,7,12,24 S, spending more time in the adjacent channel with vehicle compared to the experimental channel, after accounting for their side biases before odorant application. Analogs with 3 sulfate groups regardless of their position at C-3, C-7, C-12, or C-24 (PZ-3,7,24 S, PZ-3,12,24 S, PZ-7,12,24 S, 3kPZ-7,12,24 S) did not induce a preference for or against the test stimuli. The addition of a fourth sulfate group induced aversion (PZ-3,7,12,24 S; *P* < 0.001). With the exception of PZ-12,24 S and PZ-3,7,12,24 S, the compounds that induced preference or aversion also elicited normalized EOG response magnitudes greater than 2.

In addition to characterizing the behavioral preference for each analog, we also assessed female behavioral responses to each analog mixed with 3kPZS (1:1, each at 10^− 12^ M) to determine if any analogs acted as 3kPZS behavioral antagonists that abated ovulated female preference for 3kPZS in a flume (Fig. 3; Table 1). Females were repulsed from a mixture of 3kPZS with either PZ-3,7,12,24 S, PZ, or PZ-7,12,24 S (*P* < 0.05). 3kPZ-7,12,24 S, PZ-3,12,24 S, PZS, PZ-12,24 S, and PZ-3,7,24 S neutralized female attraction to 3kPZS (*P* > 0.05). PZ-3,24 S, 3kPZ, and PZ-7,24 S did not appear to influence female attraction to 3kPZS as they retained a preference for 3kPZS in the presence of these analogs (*P* < 0.05). When comparing the structure of 11 analogs tested to the behavioral response induced when mixed with 3kPZS, we found analogs with a hydroxyl at C-3 and C-7 and sulfate at C-24 neutralized female attraction to 3kPZS (PZS and PZ-12,24 S). For pairs of analogs sharing the same functional group at C-7, C-12, C-24, those with a hydroxyl at C-3 antagonized 3kPZS responses more than those with a ketone at C-3 (PZS vs. 3kPZS; PZ vs. 3kPZ; PZ-7,12,24 S vs. 3kPZ-7,12,24 S). Bile salts with more sulfate groups tend to disrupt ovulated female attraction to 3kPZS. Analogs with at least 3 sulfates across C-3, C-7, C-12, or C-24 (3kPZ-7,12,24 S, PZ-3,12,24 S, PZ-3,7,24 S, PZ-7,12,24 S, PZ-3,7,12,24 S) either neutralized or repulsed females from 3kPZS.

## Discussion

This study expands our understanding of sea lamprey chemoreception by elucidating the structure-activity relationships of bile acid derivatives and evaluating their capacity to modulate responses to the male sex pheromone 3kPZS. Our findings demonstrate that sulfate substitutions at specific positions within the bile acid scaffold alter olfactory responses and subsequent behavioral attraction to 3kPZS, providing additional insights into how functional group substitutions shape chemosensory responses. Specifically, we found that systematic modifications to bile acid sulfation at C-3, C-7, C-12, and/or C-24 on bile acids influence the olfactory and behavioral responses of sea lamprey. This builds on a prior study that screened commercially available compounds and identified general structural motifs associated with enhanced activity as 3kPZS olfactory inhibitors, including a steroid ring, negatively charged oxygen in positions equivalent to the terminal C-24 sulfate oxygens in 3kPZS, or an alkyl sulfate side chain (Raschka et al. 2018). Based on results from Raschka et al. 2018, we designed a series of analogs in which sulfate groups were introduced at various combinations of the C-3, C-7, C-12, and C-24 positions. This approach allowed us to isolate the specific contribution of these modifications, refining our understanding of how ligand structure governs olfactory sensitivity and behavioral outcomes. Our results build upon previous research showing that bile acid derivatives, particularly those with a C-24 sulfate on the side chain, can modulate the detection of the male-released sex pheromone 3kPZS based on electrophysiology recordings and behavioral responses (Raschka et al. 2018; Buchinger et al. 2020; Scott et al. 2023).

Our concentration-response recordings reveal consistent structure-activity patterns between electro-olfactogram recordings, which reflect the summation of generator potentials of olfactory sensory neurons, and the activation of sea lamprey odorant receptors OR320a and OR320b by 3kPZS and its analogs as characterized in a previous study (Zhang et al. 2020). Zhang and colleagues (2020) de-orphanized two highly related sea lamprey odorant receptors, OR320a and OR320b, in the olfactory epithelium, which were activated by 3kPZS and PZS, as well as other bile acid analogs but to a lesser extent. We observed several parallel structure-activity relationships in the activation of OR320a and OR320b by C_24_ 5α-bile acids (Zhang et al. 2020) and our electro-olfactogram recordings on the main olfactory epithelium when stimulated with 3kPZS and its analogs. Specifically, we found: (1) 3kPZS and PZS were the most potent ligands; (2) Other bile acids analogs acted as partial agonists that stimulated responses but with smaller response magnitudes; (3) The presence of a C-12 hydroxyl and C-24 sulfate on C_24_ 5α-bile acids were critical for inducing potent EOG responses. More specifically, among analogs sharing identical functional groups at C-3, C-7, and C-24, those with a C-12 hydroxyl group typically induced larger magnitude responses compared to those with a C-12 sulfate group; (4) When comparing analogs that shared identical functional groups at C-3, C-7, and C-12, those with a C-24 sulfate group were more potent activators than those with a C-24 hydroxyl. In both assays, 3kPZS and PZS consistently induced larger responses than their corresponding hydroxyl analogs of 3kPZ and PZ, respectively; (5) Among analogs sharing identical functional groups at C-7, C-12, and C-24, those with a C-3 hydroxyl group induced larger responses compared to analogs with a C-3 carbonyl or a C-3 ketone group, as observed in receptor activation and based on electrophysiological assays, respectively. Given the clear trends between the electro-olfactogram responses and the receptor activation data to 3kPZS and bile acid analogs, these findings support the notion that at least part of the electrophysiological responses are mediated by OR320a and OR320b. Further investigation into the molecular mechanisms underlying the effects of these analogs could provide deeper insights into the analogs’ modes of action.

The EOG results when coupled with those from behavioral assays indicate that differences in receptor activation and sensitivity likely contribute to the varied effects of the analogs on the olfactory responses and female attraction to 3kPZS. For example, adapting the olfactory epithelium to PZ-3,24 S reduced the 3kPZS electro-olfactogram response by 65%. Despite this reduction, PZ-3,24 S did not diminish female attraction to 3kPZS. In fact, females were attracted to a mixture of PZ-3,24 S and 3kPZS and PZ-3,24 S alone compared to the vehicle control in flume trials. PZ-3,24 S also elicited olfactory responses of similar magnitude to 3kPZS. These findings suggest PZ-3,24 S may function as an agonist that activates OR320a and/or OR320b and elicits olfactory and behavioral responses akin to 3kPZS. In contrast, adapting the olfactory epithelium to PZ-7,12,24 S reduced the 3kPZS electro-olfactogram response by only 35%, suggesting it shares less overlap with the 3kPZS detection mechanism than PZ-3,24 S. PZ-7,12,24 S weakly stimulated the olfactory system (normalized EOG response of 0.33) and did not induce a significant behavioral preference. However, despite its weak activation, PZ-7,12,24 S strongly suppressed female attraction to 3kPZS. Females avoided a flume channel treated with a mixture of PZ-7,12,24 S and 3kPZS. These results suggest PZ-7,12,24 S acts as a behavioral antagonist of 3kPZS and may also be a partial pharmacological antagonist of 3kPZS-induced OR320a and/or OR320b activation. Some of the bile acid analogs tested in this study appear to share olfactory receptors and/or transduction pathways with 3kPZS, while others may be detected through distinct receptor mechanisms. Our findings are consistent with previous studies on teleost and elasmobranch olfactory systems, which show these species readily discriminate between dissimilar odorants (e.g. bile acids versus amino acids) via independent receptor types, but have more limited ability to differentiate among compounds within an odorant class particularly as structure similarity increases (Michel and Derbidge 1997; Meredith et al. 2012; Laberge and Hara 2004). Michel and Derbidge (1997) found evidence based on cross-adaptation EOG recordings that the zebrafish olfactory system partially adapted to two pairs of structurally similar bile acids that differed by the presence of one hydroxyl. Adapting the olfactory epithelium to taurochenodeoxycholic acid that has 2 hydroxyls at C-3 and C-7 reduced the EOG response to taurocholic acid that has 3 hydroxyls at C-3, C-7, and C-12. Additionally, adapting to glycochenodeoxycholic acid with 2 hydroxyls C-3 and C-7 reduced responses to glycocholic acid with 3 hydroxyls at C-3, C-7, and C-12, suggesting these structurally similar bile acids are likely detected by overlapping receptor mechanisms. The combined olfactory responses and behavioral outcomes suggest that both overlapping and differential receptor activation play a role in the nuanced olfactory detection and discrimination of bile acids.

Further research is needed to validate the behavioral influence of these analogs on female attraction to 3kPZS in a natural stream to better understand the utility of behavioral antagonists as a potential tactic to manage invasive sea lamprey in the Laurentian Great Lakes Basin. The laboratory-like flume assays used a controlled experimental design that facilitated direct comparison of the analogs on female attraction to 3kPZS. However, flume assays do not fully replicate all environmental, social, and physiological contexts associated with natural river systems that can modulate expression of odor-mediated behaviors (Johnson and Li 2010), underscoring the importance of confirming flume results under natural field conditions. Future research should prioritize testing bile acid analogs that successfully antagonized 3kPZS responses in this study in a large-scale field setting to determine the efficacy of analogs in influencing sea lamprey movement and spawning behaviors in more dynamic aquatic environments. More research is needed to refine and optimize the structural features of these analogs, particularly those that neutralized or reversed 3kPZS attraction and reduced 3kPZS olfactory responses, to enhance their efficacy. By systematically modifying substituents of bile acid scaffolds, synthesizing them, and evaluating their effects in an iterative manner, it may be possible to design more effective compounds that selectively disrupt pheromone-induced responses.

## Conclusions

In conclusion, we have shown that specific functional group modifications of C_24_ 5α-bile acids result in analogs that either mimic or interfere with pheromone-induced signaling, providing additional understanding into the key structural determinants for ligand-olfactory receptor interactions that influence subsequent chemosensory responses. Our findings offer insights into the development of behavioral antagonists to disrupt pheromone-mediated mate search for invasive sea lamprey in the Laurentian Great Lakes.

## Supplementary Information

Below is the link to the electronic supplementary material.

Supplementary Material 1

## Data Availability

The data that support the findings of this study are available in the supplementary material of this article.
